# Supplementary material for: PhySortR: a fast, flexible tool for sorting phylogenetic trees in R
Source: PeerJ. 2016 May 12;4:e2038. doi: 10.7717/peerj.2038 (PMC4868591; doi:10.7717/peerj.2038)
Supplement: Text S1 [file peerj-04-2038-s004.docx]

Supplementary Text S1

Installation notes for PhySortR

PhySortR is available on the Comprehensive R Archive Network (CRAN): <https://cran.r-project.org/web/packages/PhySortR/>. PhySortR depends on the commonly used phylogenetics packages in R, *ape* and *phytools*. All these packages can be installed directly from the CRAN in the R environment, and should work fine with R version 3.0 and above. If *ape* and *phytools* are not installed, one may use the following instructions in R to install them (steps 1-3 below), before installing PhySortR (step 4).

**# 1. Install ape from CRAN**

install.packages("ape")

**# 2. Install Biostrings from Bioconductor (required for phytools)**

source("https://bioconductor.org/biocLite.R")

biocLite("Biostrings")

**# 3. Install phytools from CRAN**

install.packages("phytools")

**# 4. install PhySortR from CRAN**

install.packages("PhySortR")

One may install PhySortR (step 4) directly if *ape* and *phytools* were already installed. The three packages can then be loaded (step 5) as follows:

**# 5. load ape, phytools and phySortR**

library(ape)

library(phytools)

library(PhySortR)

PhySortR functions, ***convert.eNewick*** and ***sortTrees*** will then be ready to use.
