# Supplementary material for: PhySortR: a fast, flexible tool for sorting phylogenetic trees in R
Source: PeerJ. 2016 May 12;4:e2038. doi: 10.7717/peerj.2038 (PMC4868591; doi:10.7717/peerj.2038)
Supplement: Figure S1 [file peerj-04-2038-s005.pdf]

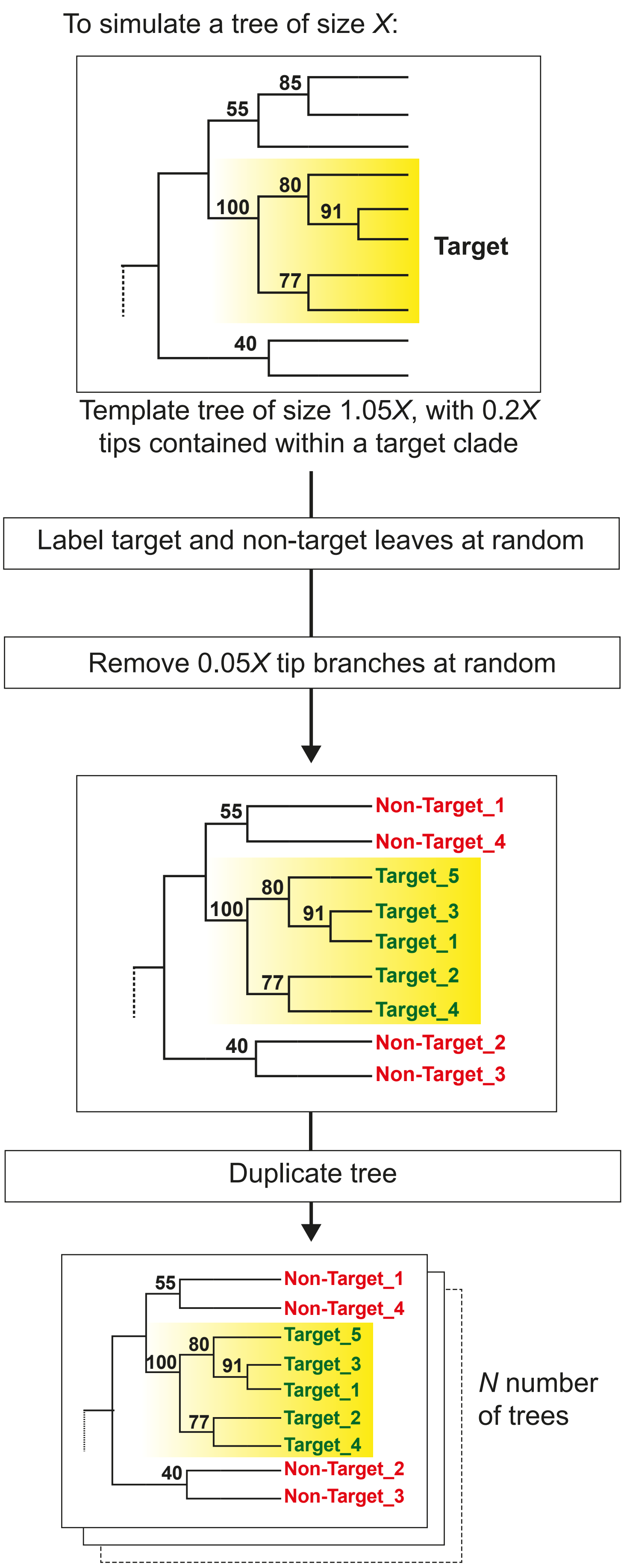

Supplementary Figure S1.
Simulation strategy for generating phylogenetic tree sets.
